# Evolutionary Dynamics of Endogenous Feline Leukemia Virus in the *Felis* Genus Through the Lens of Genomics

**DOI:** 10.1111/nyas.70278

**Published:** 2026-04-21

**Authors:** Ricardo Mouta, Liliane Tavares de Faria Cavalcante, Marcelo Alves Soares, Carlos G. Schrago, Mirela D'arc, Filipe Romero Rabelo Moreira, André Felipe Andrade dos Santos

**Affiliations:** ^1^ Department of Genetics and Graduate Program in Genetics Universidade Federal do Rio de Janeiro ‐ UFRJ Rio de Janeiro Brazil; ^2^ Oncovirology Program Brazilian National Cancer Institute ‐ INCA Rio de Janeiro Brazil

**Keywords:** bioinformatics, domestic cat, endogenous retrovirus, feline leukemia virus

## Abstract

Feline leukemia virus (*Gammaretrovirus felleu*) causes variable pathologies, partially modulated by its endogenous form (enFeLV). Although enFeLV is found in genomes of all species of the *Felis* genus, the limitations of transposable element sequencing have hindered a comprehensive characterization. In this study, we examined 17 felid genomes for enFeLV copies, confirming that enFeLV is restricted to the *Felis* genus. From the long‐read genomes of *Felis catus*, *Felis nigripes*, and *Felis chaus*, we identified 73 solo LTRs, 64 copies with genic segments, and 4 atypical copies. These were annotated and characterized using recombination assessment, phylogenetic analysis, insertion dating, and Fisher's exact tests. All enFeLV copies shared a single origin, with insertions occurring within the last 3.5 million years, except for predicted recombinants. Copy distribution, dating, and the phylogenetic position of *F. chaus* copies reinforce the hypothesis of a recent introgression of enFeLV into this species. Furthermore, 41 copies exhibit identical LTR pairs and 14 retain intact open reading frames spanning the viral genome. Uneven evolutionary pressures across the enFeLV genome were evidenced by discrepancies between tree topologies of individual genes and the preferential loss of *pol*. We conclude that enFeLV has undergone a complex post‐endogenization evolutionary history and urge additional studies of other endogenous retroviruses.

## Graphical abstract

The endogenous form of *Gammaretrovirus felleu* (feline leukemia virus; enFeLV) modulates exogenous infections. Mining of felid genomes confirmed that enFeLV is restricted to the *Felis* genus. Sequence analysis indicates retention of transcriptional and integrational capacity in *Felis catus* and introgression in the *Felis chaus* germline. The molecular record of enFeLV might be incomplete due to retrotransposition followed by loci loss, leading to a phenomenon similar to “ghost lineages.” Rather than a genomic fossil, enFeLV's activity in its hosts' genomes suggests complex evolutionary dynamics across millions of years.

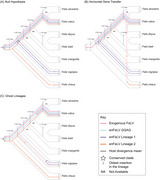

## 1 Introduction

Feline leukemia virus (FeLV; *Gammaretrovirus felleu*) infects the domestic cat (*Felis catus*), causing clinical conditions such as immunosuppression, anemia, and tumorigenesis [1]. Deadly outbreaks have erupted after spillover events into other felid species, and antibodies against the virus have been detected in other *Felis* species, although no clinical assessment is available for these cases [2, 3, 4, 5, 6]. *G. felleu* can be found integrated into the genomes of individuals belonging to the *Felis* genus [7, 8]. This form, an endogenous retrovirus (ERV), arises from the infection of germ cells followed by inactivation of the proviral genome, leading to its Mendelian inheritance as a transposable element [9]. In this regard, the endogenous form of FeLV (enFeLV) is unique because it can be expressed in *F. catus*, producing peptides and noncoding RNAs, and recombining with the exogenous form of the virus [10, 11, 12]. The most recurrent recombination event occurs in the envelope and originates FeLV‐B, a subgroup with increased oncogenic potential [11, 12].

Endogenous FeLV was first detected through DNA homology in specimens from the *Felis* genus [7]. Later, the evolutionary relationship of enFeLV copies was determined to be mostly congruent with their hosts [8]. The exception was *Felis chaus*, the earliest species to diverge within the *Felis* genus, which harbors the most recent enFeLV copies to diverge within the enFeLV clade [8, 13]. It is currently hypothesized that the original endogenization of enFeLV occurred after the divergence of *F. chaus* but before the split of *Felis nigripes*, followed by a second endogenization event after *F. nigripes* diverged [8]. This latter lineage is thought to have entered the *F. chaus* germline through hybridization with another undefined *Felis* species [8].

The challenge to sequence transposable regions such as ERVs lies in precisely assembling despite loci diversity and avoiding the formation of chimeras [14]. Long‐read sequencing reads are over 10 kbp long, which enables ERVs to be sequenced in a single read alongside its genomic context, improving detection and assembly [15]. Using publicly available genomes of *Felis* species, we conducted an in‐depth mining of enFeLV copies in their host genomes. We then annotated genes, analyzed the copies’ phylogenetic relationships, and dated the age of the insertions according to long terminal repeat (LTR) divergence. By integrating genomic evidence across multiple *Felis* species, our study updates and expands the understanding of enFeLV evolution, unraveling the dynamics of this ERV in relation to their hosts.

## 2 Materials and Methods

### 2.1 Sequence Retrieval and Dataset Compilation

We compiled two sets of genomes after searching the Ensembl, Genbank, and RefSeq databases [16, 17, 18] as of April 2024 (Table S1). The first set was composed of genomes for one species of each genus of the Felidae family. We favored chromosomal‐level assemblies, which were not available for the *Puma*, *Herpailurus*, and *Caracal* genera. The second set contained all available genomes from species of the *Felis* genus with a chromosomal‐level assembly, as well as the reference genome for *Prionailurus bengalensis*, a member of the sister genus to *Felis* [13, 19]. Each set of genomes was turned into a nucleotide database for the NCBI‐Blast+ v. 2.13.0 tool using the “makeblastdb” command and the “‐parse_seqids” flag for subsequent identification and manipulation [20, 21]. These databases are referred to as “Felidae database” and “*Felis* database” henceforth.

All five enFeLV sequences over 8000 nt long that were available in the nucleotide database of the NCBI server as of April 2024 were tested in both databases [17]. An enFeLV sequence identified by the GGAG genomic signature (accession number AY364319) was selected as the query for BLAST searches according to its highest overall similarity scores to the BLAST hits [20]. The selected sequence was segmented according to the NCBI annotation, and the whole genome, each gene, and the LTR were used independently as queries [17].

### 2.2 Scanning for enFeLVs

We used the BLASTn algorithm, which is well‐suited for detecting somewhat similar sequences such as orthologs in closely related species [20, 21]. To determine the host range of enFeLV, we queried the *gag*, *pol*, and *env* regions of the ERV against the Felidae database, applying a 50% identity as the cutoff to retain relevant hits. These relaxed parameters were selected to maximize the detection of potential enFeLV sequences in more distantly related species. For each match, we determined its genomic position using the “blastn” command and extracted 4000 base pairs of flanking regions on both sides with the “blastdbcmd” command. This process yielded a dataset of candidate enFeLV sequences for subsequent phylogenetic analysis [20, 21].

The *Felis* database was used to mine all enFeLV loci in each genome, employing multiple queries (LTR, *gag*, *pol*, *env*, and the complete genome sequence) and a cutoff of 50% coverage for high‐scoring segment pairs, which are the local alignments with no gaps that achieve the highest alignment scores in a given BLAST search [20, 21]. This strategy aimed to ensure filtering out unrelated ERVs. Match coordinates were determined using the “blastn” command, and to retrieve full‐length enFeLV sequences, 16,000 base pairs flanking regions were extracted with the “blastdbcmd” command for downstream analysis [20, 21].

### 2.3 Sequence Annotation

We annotated the enFeLV copies using the Geneious software v.2022.2.2 (https://www.geneious.com/). Stop codons and open reading frames (ORFs) were identified, and genomic regions and peptide annotations were imported from NCBI sequences AY364319 (endogenous FeLV) and NC_001940 (exogenous FeLV). A cutoff of 25% identity was established for automated annotations, followed by manual curation. In cases where individual peptides were detected, but the identity fell below the automatic threshold for the entire gene, the corresponding gene annotation was added manually. Alignments for each annotation were trimmed to retain only regions with high identity. The gene regions were plotted according to their genome of origin using the R software and the “barplot” command [22].

Mined copies were classified according to their genomic completeness. Copies containing the expected gene regions of a retrovirus were designated “typical copies,” regardless of whether they included deletions (encompassing both complete and reduced copies). Copies consisting of a single LTR and no other viral gene regions were classified as “solo LTRs.” The “atypical copies” were those fully retrieved, but exhibiting unexpected architectures, such as repetitions of the same gene. Copies that were truncated due to being near the end of an unplaced scaffold or other assembly limitations were categorized as “segments in incomplete scaffolds.” Finally, instances where two LTR segments were found contiguous to each other with no genomic viral region in between them, they were defined as “paired solo LTRs.”

The chromosomal location of each copy was annotated and visualized in a virtual karyotype in the R software using the *chromoMap* package [22]. Genomic signatures left after retroviral integration corresponding to four nucleotides upstream and downstream of the LTRs were retrieved and used to identify each individual polymorphism. Copies counts across genomes, gene counts between genes, and peptide counts within each polypeptide were compared using Fisher's exact tests with Bonferroni correction with the *rstatix* package in R software [22, 23]. All figures were prepared in Adobe Illustrator v.27.0.1 [24].

### 2.4 Recombination Analysis

To evaluate potential recombination or other similar processes between enFeLV copies, all typical copies within each genome were aligned using the Muscle v.3.8.425 program and were run through the RDP4 pipeline [25]. The detection methods used were: RDP, GENECONV, MAXCHI, CHIMAERA, RECSCAN, 3SEQ, and SISCAN. The “polish breakpoints” and “disentangle overlapping events” options were enabled, except when it initiated a processing loop, in which case the latter option was deselected before restarting the analysis, following the program manual [25]. Recombination events detected by three or more methods were removed from phylogenetic analysis, as they were considered evidence of mixed phylogenetic signals across the sequence.

### 2.5 Phylogenetic Analysis

Reference sequences of each genus of the *Orthoretrovirinae* subfamily and one representative of the *Spumaretrovirinae* subfamily [26] were used to contextualize the mined datasets (Table S1). Each dataset, together with reference sequences, was aligned using MAFFT's v.7.470 [27] “–auto” algorithm, followed by trimming using Trimal's v1.4 “‐automated1” option [28]. The ModelFinder algorithm, implemented in IQ‐TREE v2.0.3 software, was used to select the best fit nucleotide substitution model [29]. Maximum likelihood phylogenies were inferred in IQ‐TREE, with branch support assessed using 1000 ultra‐fast bootstrap replicates and 1000 SH‐aLRT iterations [29]. Approximately unbiased (AU) tests were performed in IQ‐TREE software to compare the topologies of each individual gene and the complete ERV sequence [29]. This was done to verify whether genes exhibited significantly different evolutionary histories. The resulting trees were visualized and edited in FigTree 1.4.4 and Adobe Illustrator 27.0.1 [24, 30]. All typical copies, NEWICK tree files, and the log for the AU test are available at the repository: https://github.com/Ricardo‐Mouta/2025_Evolutionary_dynamics_of_endogenous_FeLV.

### 2.6 Insertion Dating

The insertion time of each enFeLV copy was estimated using the formula *T* = *K*/2*r*, where *T* is the insertion time, *K* is the nucleotide distance between LTRs, and *r* is the mutation rate in substitution/site/year [31]. A mutation rate (*r*) of 2.3 × 10^−9^ was used, as calculated in domestic cats by Wang et al., which is consistent with rates reported in endogenous LTRs of primates [32, 33]. The nucleotide distance (*K*) between each pair of LTRs was estimated through the MEGA v.11.0.11 software [34] using the Kimura 2 parameter (K2P) nucleotide substitution model [35] applied to pairwise alignments generated with MUSCLE v.3.8.425 [36].

## 3 Results

### 3.1 Endogenous FeLV Found Exclusively in the *Felis* Genus

**FIGURE 1:**
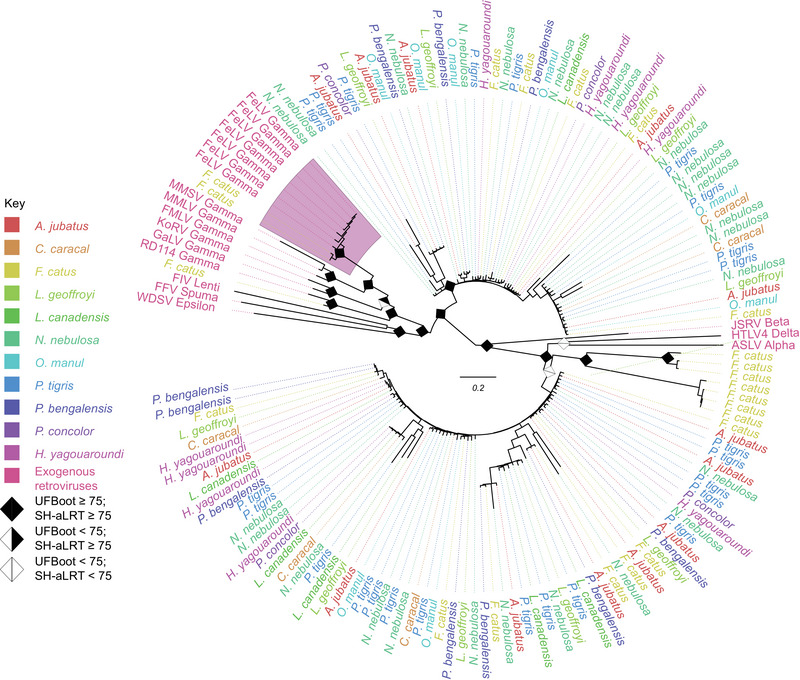
Midpoint rooted maximum‐likelihood phylogenetic tree constructed from a 3660‐nucleotide‐long alignment of 152 sequences corresponding to enFeLV's *pol* region. The inference's nucleotide substitution model was TVM with empirical base frequencies and a discrete gamma model with four rate categories. The region marked in purple contains all FeLV sequences, as well as sequences mined from the *F. catus* genome that correspond to enFeLV sequences. Node support values established through Ultrafast Bootstrap and SH‐aLRT are, respectively, included in the corresponding branch of the phylogeny. The bar scale represents a 0.2 amino acid substitutions per site per branch length equal to the bar. Table S1 contains information on each genome and reference sequence used for this analysis.

BLAST searches using the *gag* and *env* regions each yielded 10 hits, all of which were mapped exclusively to the *F. catus* genome. All 138 *pol* hits matched to the enFeLV integrase peptide (Figure 1). We assessed the phylogenetic relationships of *pol* hits retrieved from the Felidae, comprising representatives of 11 out of 14 felid genera (Table S1). A monophyletic group with maximal branch support (UFBoot = 100 / SH‐aLRT = 100) was formed between FeLV and two sequences retrieved from the *F. catus* genome, while the 136 other mined sequences were dispersed throughout the phylogeny. In this analysis, enFeLV was only found in *F. catus*, leading us to conclude that enFeLV is restricted to the genus *Felis*.

### 3.2 enFeLV as a Polymorphic ERV in the *Felis* Genus

Our strategy of querying multiple enFeLV regions in the *Felis* dataset was robust to the deletion of large segments (Table S2). The use of the high similarity pairs coverage cutoff ensured that matches represented significant portions of the sequence, in contrast to small conserved regions that can be cross‐matched from other ERVs. After removing redundant hits and extracting the flanking region from the corresponding genome, we characterized a total of 141 enFeLV copies in long‐read genomes (Figure S1). Among all enFeLV copies identified in long‐read genomes (*n* = 141), 73 were classified as solo LTRs, 64 as typical copies, and 4 as atypical copies (Table 1). No enFeLV copies were found in the outgroup *P. bengalensis*, further evidencing that enFeLV is absent in this species.

**TABLE 1:** Characterization of mined enFeLV copies in the *Felis* dataset according to the genetic completeness and genome of origin.

Long‐read genomes	Solo LTRs	Typical copies	Atypical copies	Paired solo LTRs	Segments in incomplete scaffolds	Total
Fca_9.2 (*F. catus*)	12	7	2	0	0	21
Fca_Pb1.1 (*F. catus*)	20	12	1	0	0	33
Fca_126 (*F. catus*)	19	7	1	0	0	27
Fni_SNNU (*F. nigripes*)	22	11	0	0	0	33
Fch_1.0 (*F. chaus*)	0	27	0	0	0	27
Pbe_1.1 (*P. bengalensis*)	0	0	0	0	0	0
Total	73	64	4	0	0	141
**Short‐read genomes**						
Fca_V17 (*F. catus*)	10	1	7	1	7	26
Fni_HiC (*F. nigripes*)	11	0	0	2	19	32
Total	21	1	7	3	26	58

Abbreviation: LTR, long terminal repeat.

Each *F. catus* genome contained at least one atypical enFeLV copy (Fca_126 = 1; Fca_Pb1.1 = 1; Fca_9.2 = 2; Fca_v17 = 7), whereas no atypical copies were detected in *F. nigripes* or *F. chaus*. In the Fca_126 (TGAC) and Fca_9.2 (TGAC; CAGC) genomes, atypical loci showed a repeated structural organization: two *gag* and two *env* segments were located between the LTRs and separated by an LTR‐like sequence, consistent with a duplication of the internal genomic region (Figure S1). Only the short‐read genomes, Fni_HiC and Fca_V17 (Table S1), presented the other two categories of copies (three paired solo LTRs and 26 segments in incomplete scaffolds; Table 1). Solo LTRs were found in both genomes (10 in Fca_V17 and 11 in Fni_HiC), but only a single typical copy was found in Fca_V17, as well as seven atypical copies. The typical copy that was fully assembled from the Fca_v17 was annotated, dated, and used in the phylogenies, keeping these limitations into consideration.

**FIGURE 2:**
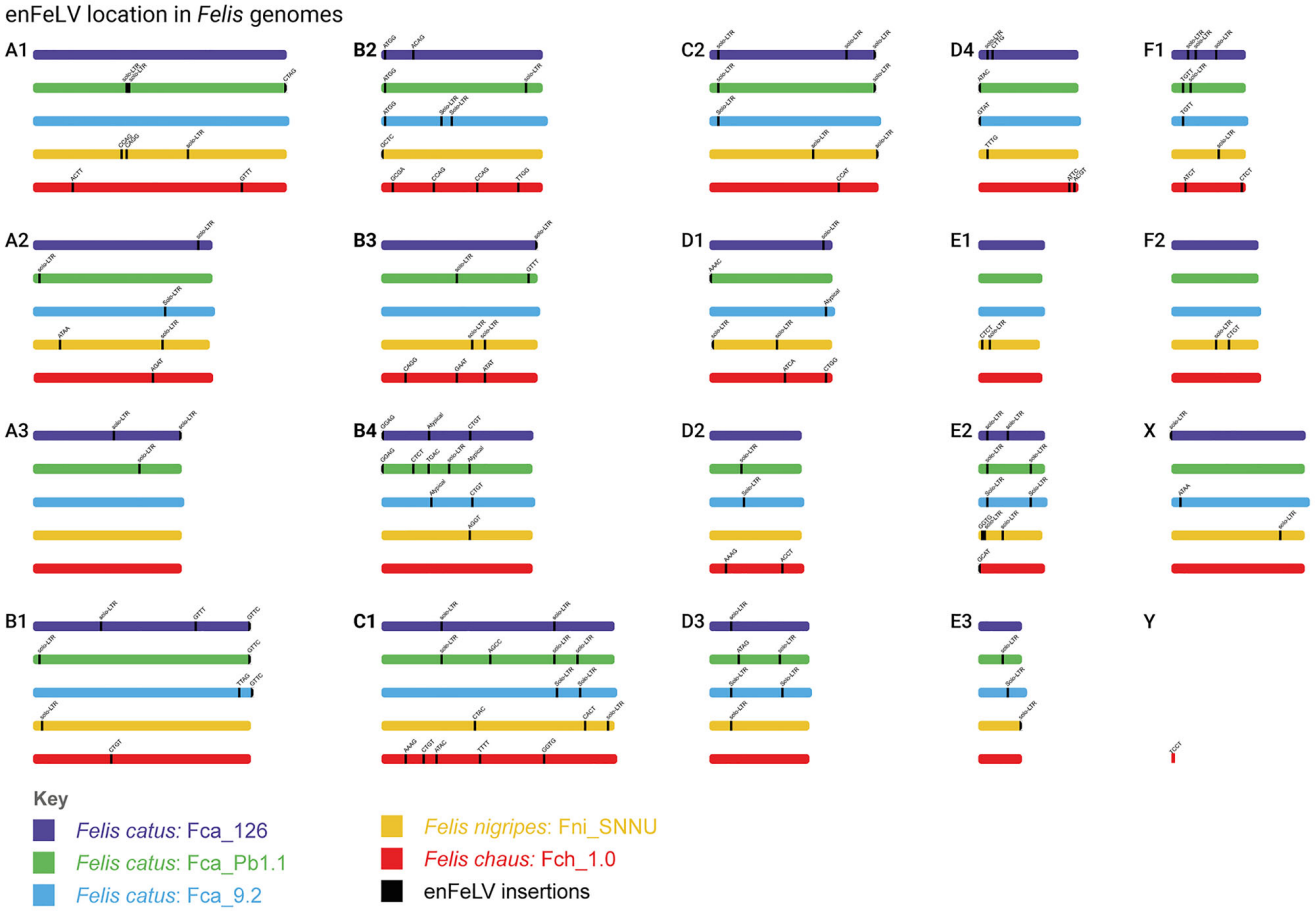
Visualization of the karyotype of the individual *Felis* genomes (depicted in different colors according to the legend in the lower left corner) divided by their homologous chromosomes, highlighting enFeLV insertion locations. Each insertion has been tagged as a solo LTR, an atypical copy, or by typical copies’ genomic signatures. Abbreviation: LTR, long terminal repeat.

The enFeLV loads in *F. catus* individuals (7−12 typical copies, 12–20 solo LTRs) were comparable to those in *F. nigripes* (11 typical copies, 22 solo LTRs; *p* = 0.958). In contrast, *F. chaus* harbored no solo LTRs and exhibited a significantly higher load of typical copies (27 total copies) compared to all other individuals (*p* < 0.001; Table 1). The chromosomal map of enFeLV showed that all chromosomes harbored enFeLV copies in at least one *Felis* individual (Figure 2). In *F. catus*, between 14 and 16 chromosomes harbored enFeLV, while 17 and 12 chromosomes held enFeLV copies in *F. nigripes* and *F. chaus*, respectively. The highest number of copies in the same chromosome was five, which was found in Fch_1.0 (C1) and Fca_Pb1.1 (B4), followed by four copies in Fca_Pb1.1 (C1) and Fch_1.0 (B2). Across all five genomes (95 chromosomes), 31 chromosomes carried one copy, 28 carried two copies, and 10 carried three copies of enFeLV, while 22 chromosomes lacked insertions entirely. No single enFeLV polymorphism was fixed across the *Felis* genus (Figure 2). However, three solo‐LTR loci were shared between *F. nigripes* and either two (chromosomes C2 and D3) or three (chromosome E2) *F. catus* genomes. Additionally, seven signatures of typical copies were present across species, although their insertion sites differed.

The 81 enFeLV copies found in the three *F. catus* long‐read genomes were distributed across 57 loci: 40 were exclusive to a single individual, 11 were shared between two genomes, and 6 were present in all three domestic cats (Figure 2). The CTGT (Fca_126 and Fca_9.2) and TGAC (Fca_Pb1.1) copies, all located in the B4 chromosome, shared loci with atypical copies in the other *F. catus* genomes. In *F. catus*, copies occupying the same locus also exhibited identical genomic signatures, with the exception of those carrying the GTTT signature. On the other hand, three pairs of *F. chaus* copies shared the same signatures (AAAG, CCAG, and CTGT) but were inserted in different loci. All copies found in *F. nigripes* had unique signatures.

### 3.3 Molecular Dynamics of enFeLV Copies: Recent Insertions and Genomic Integrity

The inferred ages of insertions, as calculated through substitutions in the LTRs of the 65 typical copies of enFeLV, indicated recent insertion events across the hosts. We estimated that 55 insertions (84.6%) occurred within the last 1 million years: 18 out of 27 *F. catus* copies (66.7%), all 27 *F. chaus* copies (100%), and 10 out of 11 (90.9%) *F. nigripes* copies (Table S2). Forty‐one copies (63.1%) presented identical LTRs, suggesting very recent insertions, although our approach was incapable of precisely dating insertion times when *K* = 0. In contrast, only 10 sequences (15.3%) were inferred to have been inserted more than 1 million years ago: three copies retrieved from the Fca_Pb1.1 (TGAC), Fca_126 (GTTC), and Fni_SNNU (TTTG) genomes (1.05 Mya each), the single typical sequence retrieved from the Fca_V17 genome (TGAC; 1.4 Mya), and six copies from the Fca_9.2 genome (ATGG, GTTC, CTAA, ATAC, TGTT, TTAT; range: 2.82−9.97 Mya).

Evidence of recombination was found in seven typical copies (10.8%), all from *F. catus* genomes (Table S2). One recombinant was found in the Fca_126 genome (CTTG; detected by three methods), one in the Fca_Pb1.1 genome (CTAG; three methods), and the Fca_9.2 genome harbored the remaining five copies (CTAA, TGTT, TTAT, ATAC, CTGT; detected by five, six, six, seven, and seven methods, respectively). These five Fca_9.2 copies included the four oldest insertions recovered across all genomes, dated between 4.28 and 9.97 Mya. Considering that estimates of recombinant copies may be biased, the oldest nonrecombinant enFeLV insertion was dated 3.5 Mya in the Fca_9.2 genome (GTTC). This locus was present in all three *F. catus* individuals (Figure 2), with corresponding insertions dated at 0.698 Mya in Fca_Pb1.1 and 1.05 Mya in Fca_126.

We identified 48 copies (73.8%) containing potentially translatable gene segments, as defined by the presence of uninterrupted ORFs across each annotated gene (Table S2 and Figure S2). Of these, 14 copies (29.2%) had uninterrupted ORFs spanning their entire length in *F. chaus* (*n* = 10) and *F. catus* (*n* = 4), but five of these (35.7%) presented deletions larger than 1000 nt, leading to truncated peptides. Additionally, 12 copies (18.5% of those with uninterrupted ORFs throughout their length) also presented identical LTRs in *F. catus* (*n* = 2) and *F. chaus* (*n* = 10), and thus, no obstructions are present in their genetic sequences for the transcription and integration into new loci.

### 3.4 Post‐Endogenization Phylogenetic Dynamics

**FIGURE 3:**
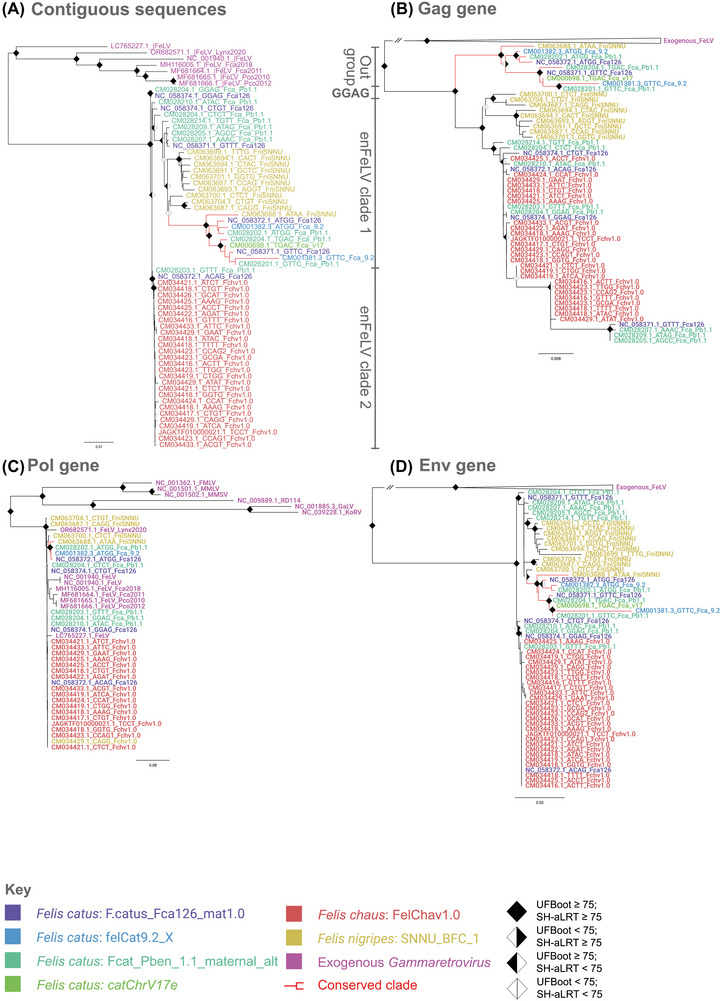
Maximum‐likelihood phylogenies of enFeLV copies, using datasets composed of the contiguous sequences (A; 8685 nucleotides), the *gag* gene (B; 1393 nucleotides), the *pol* gene (C; 1867 nucleotides), and the *env* gene (D; 1822 nucleotides). The inference's nucleotide substitution models were: TIM2 with empirical base frequencies and discrete gamma model with four rate categories (contiguous sequences); symmetric model with discrete gamma model with four rate categories (*gag* gene); TPM2 with empirical base frequencies and discrete gamma model with four rate categories (*pol* and *env* genes). Node support values established through Ultrafast Bootstrap and SH‐aLRT are, respectively, included in the corresponding branch of the phylogeny. Support values from internal nodes, as well as outer reference sequences, were omitted for viewing purposes. The bar scales represent amino acid substitutions per site per branch length equal to the bars. Table S1 contains information on each genome and reference sequence used for this analysis.

We inferred an 8685 nt long phylogeny of 77 sequences, consisting of all mined typical copies without evidence of recombination and the exogenous reference sequences (Figure 3A). Overall, the virus most similar to FeLV was *Gammaretrovirus murleu* (MLV). All mined sequences formed a monophyletic clade sister to exogenous FeLV with maximal support (100/100), indicating a single origin for enFeLV. In the endogenous clade, the first lineage to diverge was composed of the sequences that shared the GGAG signature, found in both Fca_126 and Fca_Pb1.1. Two clades followed and divided the wild species enFeLVs: one contained all *F. nigripes* sequences (enFeLV clade 1) and the other all *F. chaus* (enFeLV clade 2). The intermediate confidence values between enFeLV clades 1 and 2 might be caused by mixed phylogenetic signals, as inferred through the different topologies of the gene trees (Figure 3B−D).

EnFeLV clade 1 encompasses all of enFeLV diversity in *F. nigripes*, as well as most of *F. catus*. Each of the earliest sequences to diverge are restricted to a single genome, followed by a lineage exclusive to *F. nigripes*. The most derived lineage, emphasized by red branches in the figures, is formed by the ATAA *F. nigripes* sequence, followed by the most represented *F. catus* loci across all individuals, with both copies shared by three individuals present within this clade (ATGG, GTTC). Also, five out of six nonrecombinant copies with insertions older than a million years were also a part of this clade. For the purposes of this study, we refer to this assemblage as the “conserved clade” hereafter.

EnFeLV clade 2 is defined by a strongly supported monophyletic lineage containing all *F. chaus* sequences (95.5/100). Two *F. catus* sequences were the first and second to diverge in the clade. The first, bearing the signature GTTT in the Fca_Pb1.1 genome, is the only sequence in *F. catus* that shared its signature with another copy, but occupied a different genomic locus (81.9/99). The second, with the ACAG signature in the Fca_126 genome, was also significantly supported (95.5/100). Both sequences exceed 8.5 kb in length and have identical LTRs; only ACAG's *env* ORF was interrupted—overall strong candidates for retention of transpositional activity. The phylogenetic resolution among *F. chaus* copies was poor, probably due to the high similarity between them.

In order to explore the phylogenetic dynamics of individual genes, datasets were developed and analyzed for the *gag* (Figure 3B; 1393 nucleotides), *pol* (Figure 3C; 1867 nucleotides), and *env* (Figure 3D; 1822 nucleotides) genes. Monophyly of all enFeLV copies in relation to exogenous FeLV was evidenced in *gag* (92.9/98) and *env* (99.8/100), while in *pol*, sequences of endogenous and exogenous origin formed a mixed clade (98.9/95) with the most recent common ancestor as MLV (99.2/99). The approximately unbiased (AU) test showed significant differences between the genomic tree and all individual gene trees (*p* < 0.001; File S1). The varying tree topologies among genes document the complex evolutionary history of enFeLV in their hosts.

### 3.5 Complex Evolution of enFeLV Genes

**FIGURE 4:**
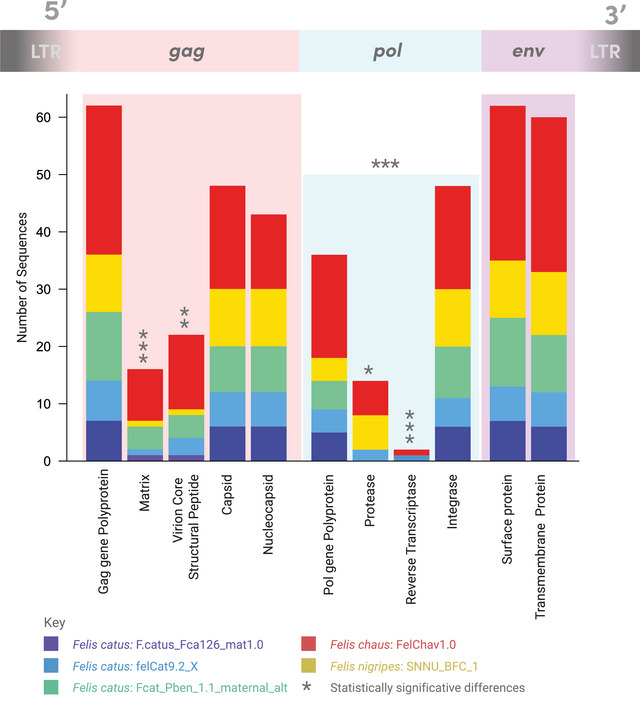
Annotation counts of genes and peptides according to their position in enFeLV's sequence, colored by the long‐read genome of origin. Asterisks (*) are used to point to the lowest significance threshold reached on counts of genes in comparison to both other genes and of peptides in comparison to all other peptides of the same gene according to the comparison's *p*‐values: *p < 0.05; **p < 0.005; ***p < 0.001.

In nonrecombinant typical copies (*n* = 65), we found a total of 65 *gag*, 50 *pol*, and 65 *env* regions (Figure 4), showing a statistically significant trend of *pol* deletion (*p* < 0.001). In both *F. catus* and *F. nigripes*, the number of *gag* and *env* sequences ranged from 7 to 12, while *pol* segments ranged from 6 to 10. In *F. chaus*, 27 copies of *gag* and 27 of *env* were found, compared to 19 fragments of *pol*. Further analysis of individual peptides revealed significant reductions in the p12 (*p* < 0.005), matrix (*p* < 0.001), protease (*p* < 0.005), and reverse transcriptase (*p* < 0.001) peptides. Across all *Felis* species, these peptides were found in fewer than 22 copies each, while all other individual peptides were found in at least 54 copies.

## 4 Discussion

In this study, we demonstrated the value of long‐read sequencing and assembly for the analysis of ERV diversity by mining enFeLVs from long‐read felid genomes. The Fca_V17 and Fni_HiC assemblies demonstrate the limitations of short‐read sequencing, which, despite presenting chromosomal level assemblies, retained enFeLV fragments in unplaced scaffolds. These findings may represent artifacts arising due to the difficulty of short‐read sequencing to resolve polymorphic loci. Notably, the Fca_V17 genome was assembled from multiple individuals, meaning that individual variation, such as ERV insertions, may not be accurately resolved unless highly conserved. The growing availability of long‐read genome sequencing enables more detailed analysis of ERVs and their roles in processes such as the placentation of mammals [37], the development of autoimmune and neurological diseases [38], and the response to infections [39], for instance. Here, we have developed and applied a genomic‐scale study of ERVs in order to establish the evolutionary history of FeLV and map the endogenous form's diversity in their host.

The first question addressed was the host range of enFeLV. Previously, ERVs were found only in the *Felis* genus, and the virus's closest relative was postulated to have infected rodents [7]; our results support this view. In all phylogenies, the virus lineage that was the immediate sister group of *G. felleu* was *G. murleu*. We found enFeLV exclusively in *Felis* species, namely, *F. chaus*, *F. nigripes*, and *F. catus*. The latter is a domesticated species with endemic FeLV infections, while the former two were the earliest species to diverge within the genus, with no known circulation of exogenous FeLV [13, 19, 40]. Genomes of the species *Felis silvestris*, *Felis libyca*, *Felis bieti*, and *Felis margarita* were not publicly available at the time of this study, which means this is still a partial image of enFeLV across its hosts.

All chromosomes harbored enFeLV in at least one individual, corroborating with previous studies [41]. In both *F. catus* and *F. nigripes*, enFeLV loads were comparable to previous qPCR or fluorescent in‐situ hybridization assays [39, 42, 43], but were discrepant from the loads found by a targeted‐enrichment approach [41]. Further studies are required to assess best practices for ERV loci determination. Endogenous FeLV sequences were polyphyletic in *F. catus* and *F. nigripes*, and solo‐LTR loci are possibly shared among species. On the other hand, in *F. chaus*, load distribution and phylogenetic positioning were divergent from those of the other species. The position of the monophyletic group formed by all of its enFeLV copies, as well as the absence of solo LTRs, is compatible with a recent origin of the ERV in this species [9], contrasting with its position in the host phylogeny [19].

EnFeLV phylogenetic diversity is structured into two major lineages, consistent with the previous analysis by Polani et al. using 3’ LTR and partial *env* sequences [8]. Their study, however, captured a limited aspect of enFeLV evolution. Here, the inclusion of multiple individuals and additional genes revealed incomplete lineage sorting and substantially improved phylogenetic resolution [44]. Polani et al. [8] also postulated that an introgression event was responsible for introducing enFeLV in *F. chaus*, which explains both its recent origin in the species and its similarity with copies found in *F. catus*. Although further studies are required to establish the donor species, ABBA‐BABA tests point that *F. margarita* is the most likely source of hybridization with *F. chaus* [19]. Additionally, the high similarity, genetic completeness, and insertion datings of *F. chaus* sequences are coherent with a rapid process of mobilization and establishment of transposable elements across the genome following the introgression—a process known as genomic shock [45].

In order to have a stronger basis of comparison between enFeLV integration in the *F. chaus* genome and its divergence from the genus, we have considered the confidence intervals for both the mutation rate and divergence time of the species. Considering that the estimate of the yearly mutation rate ranges from 2e‐9 to 2.5e‐9 [32, 33], we established the integration of enFeLV to have occurred between 320,513 and 801,284 years ago. The minimum divergence time of the split of *F. chaus* within the *Felis* genus is 2 million years ago [19]. Therefore, it is still incompatible with the maximum integration time for enFeLV.

The *F. chaus* introgression hypothesis is an example of the potential for non‐Mendelian inheritance in enFeLV. The evolutionary history of enFeLV is complex and nonhomogeneous along its genome. Certain segments appear to have been preferentially deleted, and *F. catus* enFeLV copies may have recombined among themselves, generating mixed phylogenetic signals. This is particularly evident in Fca_9.2, where multiple results diverged from those of other genomes. For instance, we found the six oldest insertion dates and evidence of recombination in five out of seven of its typical copies. While such patterns could theoretically arise from sequencing or assembly artifacts, we found no evidence to support this scenario. Instead, they may reflect evolutionary processes specific to the sequenced individual's Abyssinian breed, which notably exhibits the highest prevalence of exogenous FeLV infection in Britain [46].

**FIGURE 5:**
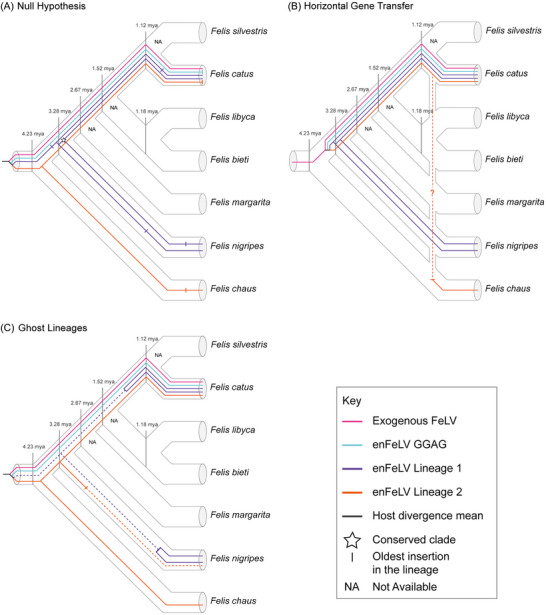
Hypothesis trees representing the dynamics of enFeLV lineages across their host genomes. (A) Null hypothesis, considering that no other process except Mendelian inheritance is present. (B) Alternative hypothesis 1, considering the presence of horizontal gene transfer throughout enFeLV's evolutionary history. (C) Alternative hypothesis 2, considering that not all loci in enFeLV's evolutionary history are available, similar to ghost lineages in the fossil record. Mean divergence of each host lineage data used from Li et al. [19]. “NA” represents genomes that were not available at the time of the study.

We further examined alternative inheritance hypotheses observed in enFeLV (Figure 5). The null hypothesis (Figure 5A) assumes strictly Mendelian inheritance. However, this model fails to account for certain patterns, such as the distribution of multiple enFeLV Clade 1 lineages in both *F. nigripes* and *F. catus* and the absence of enFeLV Clade 2 in *F. nigripes*. Our first alternative hypothesis (Figure 5B) proposes that horizontal gene transfer has happened at least once within enFeLV's host range, resulting in mixed inheritance patterns among species. The potential introgression in *F. chaus* is the clearest example. The second alternative hypothesis (Figure 5C) suggests that even though specific loci might be lost, enFeLV lineages that perform retrotransposition persist, leaving an incomplete molecular record, a process similar to ghost lineages in the fossil record [47, 48]. These hypotheses are not mutually exclusive, as introgression could occur in lineages that later become extinct [48].

The approximate ages for every divergence in the *Felis* genus have been established by previous phylogenomic studies [19]. When this timescale is compared to the timing of the enFeLV insertion as estimated by LTR divergence, the insertion of all typical copies detected in *F. catus* are younger than the age of the common ancestor of the *F. silvestris* complex (Figure 5A); the only exceptions are copies of the Fca_9.2 genome. *F. silvestris* genomes are necessary to further validate this finding. A similar pattern is observed in *F. chaus* and *F. nigripes*: in both species, the insertion ages of all typical copies postdate the divergence of their respective host lineages. Although heterozygosity for enFeLV has been previously observed in *F. catus* [42], we were unable to verify this hypothesis as the genomes used in this study are haploid assemblies. Although a minority of solo‐LTR loci were shared between *F. catus* and *F. nigripes*, most of enFeLV's diversity is exclusive to each individual. This indicates the active spread of enFeLV in its hosts in the last million years.

The equation used to date ERV insertions evidently relies on several assumptions that affect the accuracy and precision of the enFeLV timescale. For instance, we assume that the mutation rate of ERVs is stable, and they are subject to neutral evolution [31]. None of these assumptions can be applied to enFeLV with certainty. This ERV still retains remarkable genomic integrity, despite its endogenization event taking place at least 3 million years ago. Furthermore, the assumption of neutral evolution is also debatable, considering that enFeLV modulates the infections of the exogenous counterpart [39], and that specific segments of the genome are preferentially lost. Novel methods that take into account those characteristics are needed to precisely date transcriptionally active ERVs.

We found that some ORFs were preferentially lost in enFeLV. The most frequent were the matrix protein p15 and the RNA‐binding phosphoprotein p12 in the 5′ region of the *gag* gene, and the reverse transcriptase and protease peptides in the *pol* gene. It is interesting to note that these are also the regions most commonly flagged as recombinant in other copies (data not shown). All those proteins play roles in the early stages of infection in exogenous retroviruses, either in the maturation of the viral particle or before the integration of the viral sequence into the host genome [9, 26]. This pattern is congruent with either a survivorship bias of natural selection, meaning that copies that lack these segments are more likely to be kept in the gene pool, or directed inactivation of ERVs by host genomic maintenance pathways.

The canonical idea of ERVs as inactivated retroviruses is challenged by our findings. Potential for retrotransposition, biased deletions, and uneven phylogenetic signals between viral genes were evidenced in enFeLV. EnFeLV sequences have lost specific gene fragments in a nonrandom fashion. We hypothesize that either natural selection favoring viruses with those changes or host‐mediated mutagenesis are responsible for this phenomenon. Either through host interference or natural selection, enFeLV has likely been subject to forces other than pure random genetic drift. Future studies will determine the relative impact of specific loci to the dynamics of their host and exogenous counterparts, as well as the role of different evolutionary forces such as natural selection and recombination in the diversification of enFeLV across its host range. Our methodological framework can be applied to investigate other recently endogenized retroviruses, providing a means to assess whether the evolutionary patterns observed here are general features of ERV evolution.

## 5 Conclusion

By mining long‐read felid genomes, we show that enFeLV is restricted to the genus *Felis* and derives from a single endogenization event followed by a complex post‐integration history. Most enFeLV insertions are recent, with many retaining intact genomic features, including identical LTRs and uninterrupted ORFs, consistent with ongoing retrotranspositional potential. EnFeLV diversity is highly polymorphic: most loci are individual‐specific, no insertion is fixed across *Felis*, and shared loci between species are rare. Phylogenetic and dating analyses support non‐Mendelian inheritance processes, most notably introgression into *F*. *chaus*, and reveal heterogeneous evolutionary pressures across the viral genome, including preferential loss of *pol* and discordant gene histories. Together, these findings demonstrate that enFeLV is not a passive genomic relic but a dynamic ERV shaped by recent activity, lineage‐specific processes, and interactions with both host genomes and exogenous FeLV. Comprehensive long‐read genomic sampling and functional studies will be necessary to fully resolve the evolutionary and biological consequences of enFeLV in felids.

## Author Contributions

Ricardo Mouta: Designed research, performed research, analyzed data, and wrote the paper. Liliane Tavares de Faria Cavalcante: Designed research and analyzed data. Marcelo Alves Soares: Analyzed data and wrote the paper. Carlos G. Schrago: Contributed new analytical tools, analyzed data, and wrote the paper. Mirela D'arc: Analyzed data and wrote the paper. Filipe Romero Rabelo Moreira: Contributed new analytical tools, analyzed data, and wrote the paper. André Felipe Andrade dos Santos: Designed research, analyzed data, and wrote the paper.

## Conflicts of Interest

The authors declare that there are no conflicts of interest.

## Supporting information




**Supplementary Material**: nyas70278‐sup‐0001‐SuppMat‐SF1.pdf

**Supplementary Material**: nyas70278‐sup‐0001‐SuppMat‐SF2.pdf

**Supplementary Material**: nyas70278‐sup‐0001‐SuppMat‐ST1.pdf

**Supplementary Material**: nyas70278‐sup‐0001‐SuppMat‐ST2.pdf

## References

[1] W. F. H. Jarrett , E. M. Crawford , W. B. Martin , and F. Davie , “Leukæmia in the Cat: A Virus‐Like Particle Associated With Leukæmia (Lymphosarcoma).” Nature 1964;202(4932):567–568, https://pubmed.ncbi.nlm.nih.gov/14195054/. 14195054 10.1038/202567a0

[2] M. L. Meli , V. Cattori , F. Martínez , G. López , A. Vargas , M. A. Simón , et al., “Feline Leukemia Virus and Other Pathogens as Important Threats to the Survival of the Critically Endangered Iberian lynx (*Lynx pardinus*).” PLoS ONE 2009;4(3):e4744, https://dx.plos.org/10.1371/journal.pone.0004744. 19270739 10.1371/journal.pone.0004744 PMC2649436

[3] E. S. Chiu , S. Kraberger , M. Cunningham , L. Cusack , and M. Roelke , and S. Vandewoude , “Multiple Introductions of Domestic Cat Feline Leukemia Virus in Endangered Florida Panthers.” Emerging Infectious Diseases 2019;25(1):92–101, https://pmc/articles/PMC6302599/. 30561312 10.3201/eid2501.181347 PMC6302599

[4] M. J. Daniels , M. C. Golder , O. Jarrett , and D. W. MacDonald , “Feline Viruses in Wildcats From Scotland.” Journal of Wildlife Diseases 1999;35(1):121–124, 10.7589/0090-3558-35.1.121. 10073361

[5] C. M. Leutenegger , R. Hofmann‐Lehmann , C. Riols , M. Liberek , G. Worel , P. Lups , et al., “Viral Infections in Free‐Living Populations of the European Wildcat.” Journal of Wildlife Diseases 1999;35(4):678–686, https://pubmed.ncbi.nlm.nih.gov/10574526/. 10574526 10.7589/0090-3558-35.4.678

[6] S. Ostrowski , M. Van Vuuren , D. M. Lenain , and A. Durand , “A Serologic Survey of Wild Felids From Central West Saudi Arabia.” Journal of Wildlife Diseases 2003;39(3):696–701, 10.7589/0090-3558-39.3.696. 14567233

[7] R. E. Benveniste , C. J. Sherr , and G. J. Todaro , “Evolution of Type C Viral Genes: Origin of Feline Leukemia Virus.” Science 1975;190(4217):886–888, https://www.sciencemag.org/lookup/doi/10.1126/science.52892. 52892 10.1126/science.52892

[8] S. Polani , A. L. Roca , B. B. Rosensteel , S. O. Kolokotronis , and G. K. Bar‐Gal , “Evolutionary Dynamics of Endogenous Feline Leukemia Virus Proliferation Among Species of the Domestic Cat Lineage.” Virology 2010;405(2):397–407, 10.1016/j.virol.2010.06.010. 20615520

[9] D. L. Mager and J. P. Stoye . Mammalian Endogenous Retroviruses. In Michael Chandler , Martin Gellert , Alan M. Lambowitz , Phoebe A. Rice , Suzanne B. Sandmeyer (Eds.), Mobile DNA III. ASM Press; 2015 1079–1100, http://doi.wiley.com/10.1128/9781555819217.ch47.

[10] M. A. Stewart , M. Warnock , A. Wheeler , N. Wilkie , J. I. Mullins , D. E. Onions , et al., “Nucleotide Sequences of a Feline Leukemia Virus Subgroup A Envelope Gene and Long Terminal Repeat and Evidence for the Recombinational Origin of SubGroup B Viruses.” Journal of Virology 1986;58(3):825–834, https://journals.asm.org/journal/jvi. 3009890 10.1128/jvi.58.3.825-834.1986 PMC252989

[11] A. S. McDougall , A. Terry , T. Tzavaras , C. Cheney , J. Rojko , and J. C. Neil , “Defective Endogenous Proviruses Are Expressed in Feline Lymphoid Cells: Evidence for a Role in Natural Resistance to Subgroup B Feline Leukemia Viruses.” Journal of Virology 1994;68(4):2151–2160, https://journals.asm.org/journal/jvi. 8138999 10.1128/jvi.68.4.2151-2160.1994 PMC236690

[12] E. S. Chiu and S. Vandewoude , “Endogenous Retroviruses Drive Resistance and Promotion of Exogenous Retroviral Homologs.” Annual Review of Animal Biosciences 2021; 9: 225–248, 10.1146/annurev-animal-050620. 33290087

[13] A. Kitchener , C. Breitenmoser , E. Eizirik , A. Gentry , L. Werdelin , A. Wilting , et al. A Revised Taxonomy of the Felidae. The Final Report of the Cat Classification Task Force of the IUCN/SSC Cat Specialist Group. 2017 [cited 2023 Nov 20]. http://repository.si.edu/xmlui/handle/10088/32616.

[14] S. Shahid and R. K. Slotkin . (2020), “The Current Revolution in Transposable Element Biology Enabled by Long Reads.” Current Opinion in Plant Biology 54, 49–56, 10.1016/j.pbi.2019.12.012. 32007731

[15] E. Debladis , C. Llauro , M. C. Carpentier , M. Mirouze , and O. Panaud . (2017), “Detection of Active Transposable Elements in *Arabidopsis thaliana* Using Oxford Nanopore Sequencing Technology.” BMC Genomics [Electronic Resource] 18(1), 537, 10.1186/s12864-017-3753-z. 28715998 PMC5513335

[16] F. Cunningham , J. E. Allen , J. Allen , J. Alvarez‐Jarreta , M. Ridwan Amode , I. M. Armean , et al., “Ensembl 2022. Database Issue.” Nucleic Acids Research 2022;50:989, 10.1093/nar/gkab1049.35018464

[17] E. W. Sayers , J. Beck , J. R. Brister , E. E. Bolton , K. Canese , D. C. Comeau , et al., “Database Resources of the National Center for Biotechnology Information.” Nucleic Acids Research 2020;48(D1):D9–16, https://pubmed.ncbi.nlm.nih.gov/31602479/. 31602479 10.1093/nar/gkz899 PMC6943063

[18] N. A. O'Leary , M. W. Wright , J. R. Brister , S. Ciufo , D. Haddad , R. McVeigh , et al., “Reference Sequence (RefSeq) Database at NCBI: Current Status, Taxonomic Expansion, and Functional Annotation.” Nucleic Acids Research 2016;44(D1):D733–D745, http://www.ncbi.nlm.nih.gov/books/. 26553804 10.1093/nar/gkv1189 PMC4702849

[19] G. Li , B. W. Davis , E. Eizirik , and W. J. Murphy , “Phylogenomic Evidence for Ancient Hybridization in the Genomes of Living Cats (Felidae).” Genome Research 2016;26(1):1–11, http://www.genome.org/cgi/doi/10.1101/gr.186668.114. 26518481 10.1101/gr.186668.114 PMC4691742

[20] S. F. Altschul , W. Gish , W. Miller , E. W. Myers , and D. J. Lipman , “Basic Local Alignment Search Tool.” Journal of Molecular Biology 1990;215(3):403–410, https://pubmed.ncbi.nlm.nih.gov/2231712/. 2231712 10.1016/S0022-2836(05)80360-2

[21] BLAST® Command Line Applications User Manual. National Center for Biotechnology Information (US); 2008, https://www.ncbi.nlm.nih.gov/books/NBK279690/.

[22] R Core Team . R: The R Project for Statistical Computing [Internet]. R‐project.org. 2022, https://www.r‐project.org.

[23] D. G. Altman . Practical Statistics for Medical Research. Chapman & Hall; 1991.

[24] Adobe Inc . Adobe Illustrator. 2019, https://adobe.com/products/illustrator.

[25] D. P. Martin , B. Murrell , M. Golden , A. Khoosal , and B. Muhire , “RDP4: Detection and Analysis of Recombination Patterns in Virus Genomes.” Virus Evolution 2015;1(1), vev003, 10.1093/ve/vev003. 27774277 PMC5014473

[26] J. P. Stoye , J. Blomberg , J. M. Coffin , H. Fan , B. Hahn , J. Neil , et al. Retroviridae—Reverse Transcribing DNA and RNA Viruses—Reverse Transcribing DNA and RNA Viruses (2011)—International Committee on Taxonomy of Viruses (ICTV). 2011 [cited 2021 Mar 15]. https://talk.ictvonline.org/ictv‐reports/ictv_9th_report/reverse‐transcribing‐dna‐and‐rna‐viruses‐2011/w/rt_viruses/161/retroviridae.

[27] K. Katoh , K. Misawa , K. I. Kuma , and T. Miyata , “MAFFT: A Novel Method for Rapid Multiple Sequence Alignment Based on Fast Fourier Transform.” Nucleic Acids Research 2002;30(14):3059–3066, https://pmc/articles/PMC135756/. 12136088 10.1093/nar/gkf436 PMC135756

[28] S. Capella‐Gutiérrez , J. M. Silla‐Martínez , and T. Gabaldón , “trimAl: A Tool for Automated Alignment Trimming in Large‐Scale Phylogenetic Analyses.” Bioinformatics 2009;25(15):1972–1973, https://academic.oup.com/bioinformatics/article/25/15/1972/213148. 19505945 10.1093/bioinformatics/btp348 PMC2712344

[29] L. T. Nguyen , H. A. Schmidt , A. von Haeseler , and B. Q. Minh , “IQ‐TREE: A Fast and Effective Stochastic Algorithm for Estimating Maximum‐Likelihood Phylogenies.” Molecular Biology and Evolution 2015;32(1):268–274, https://academic.oup.com/mbe/article/32/1/268/2925592. 25371430 10.1093/molbev/msu300 PMC4271533

[30] A. Rambaut . (2018). FigTree. [online] Ed.ac.uk. Available at: http://tree.bio.ed.ac.uk/software/figtree/.

[31] P. SanMiguel , B. S. Gaut , A. Tikhonov , Y. Nakajima , and J. L. Bennetzen , “The Paleontology of Intergene Retrotransposons of Maize.” Nature Genetics 1998;20(1):43–45, http://genetics.nature.com. 9731528 10.1038/1695

[32] R. J. Wang , M. Raveendran , R. A. Harris , W. J. Murphy , L. A. Lyons , J. Rogers , et al., “De Novo Mutations in Domestic Cat Are Consistent With an Effect of Reproductive Longevity on Both the Rate and Spectrum of Mutations.” Molecular Biology and Evolution 2022;39(7), msac147, https://pubmed.ncbi.nlm.nih.gov/35771663/. 35771663 10.1093/molbev/msac147 PMC9290555

[33] W. E. Johnson and J. M. Coffin , “Constructing Primate Phylogenies From Ancient Retrovirus Sequences.” Proceedings of the National Academy of Sciences 1999;96(18):10254–10260, https://www.pnas.org/doi/abs/10.1073/pnas.96.18.10254. 10.1073/pnas.96.18.10254 PMC17875 10468595

[34] K. Tamura , G. Stecher , and S. Kumar , “MEGA11: Molecular Evolutionary Genetics Analysis Version 11.” Molecular Biology and Evolution 2021;38(7):3022–3027, http://creativecommons. 33892491 10.1093/molbev/msab120 PMC8233496

[35] M. Kimura , “A Simple Method for Estimating Evolutionary Rates of Base Substitutions Through Comparative Studies of Nucleotide Sequences.” Journal of Molecular Evolution 1980;16(2):111–120, 10.1007/BF01731581. 7463489

[36] R. C. Edgar , “MUSCLE: Multiple Sequence Alignment With High Accuracy and High Throughput.” Nucleic Acids Research 2004;32(5):1792–1797, 10.1093/nar/gkh340. 15034147 PMC390337

[37] C. Lavialle , G. Cornelis , A. Dupressoir , C. Esnault , O. Heidmann , C. Vernochet , et al., “Paleovirology of “Syncytins”, Retroviral Env Genes Exapted for a Role in Placentation.” Philosophical Transactions of the Royal Society B: Biological Sciences 368: 2013, 1850–1855, https://royalsocietypublishing.org/doi/abs/10.1098/rstb.2012.0507. 10.1098/rstb.2012.0507 PMC3758191 23938756

[38] M. Suntsova , A. Garazha , A. Ivanova , D. Kaminsky , A. Zhavoronkov , and A. Buzdin , “Molecular Functions of Human Endogenous Retroviruses in Health and Disease.” Cellular and Molecular Life Sciences 2015;72(19):3653–3675, https://link.springer.com/article/10.1007/s00018‐015‐1947‐6. 26082181 10.1007/s00018-015-1947-6 PMC11113533

[39] J. A. Powers , E. S. Chiu , S. J. Kraberger , M. Roelke‐parker , I. Lowery , K. Erbeck , et al., “Feline Leukemia Virus (FeLV) Disease Outcomes in a Domestic Cat Breeding Colony: Relationship to Endogenous FeLV and Other Chronic Viral Infections.” Journal of Virology 2018;92(18):1–16, 10.1128/JVI.00649-18. PMC6146681 29976676

[40] W. D. Hardy , A. J. McClelland , E. G. MacEwen , P. W. Hess , A. A. Hayes , and E. E. Zuckerman , “The Epidemiology of the Feline Leukemia Virus (FeLV).” Cancer 1977;39(4 S):1850–1855, https://acsjournals.onlinelibrary.wiley.com/doi/full/10.1002/1097‐0142%28197704%2939%3A4%3C1850%3A%3AAID‐CNCR2820390817%3E3.0.CO%3B2‐S. 192437 10.1002/1097-0142(197704)39:4+<1850::aid-cncr2820390817>3.0.co;2-s

[41] E. S. Chiu , C. A. McDonald , R. B. Gagne , H. Dunkleberger , M. Moxcey , and S. VandeWoude . (2024), “Endogenous Feline Leukemia Virus Long Terminal Repeat Integration Site Diversity Is Highly Variable in Related and Unrelated Domestic Cats.” Retrovirology 21(1), 3, 10.1186/s12977-024-00635-0. 38347535 PMC10863107

[42] A. L. Roca , W. G. Nash , J. C. Menninger , W. J. Murphy , and S. J. O'Brien , “Insertional Polymorphisms of Endogenous Feline Leukemia Viruses.” Journal of Virology 2005;79(7):3979–3986, https://journals.asm.org/doi/10.1128/JVI.79.7.3979‐3986.2005?url_ver=Z39.88‐2003&rfr_id=ori:rid:.org&rfr_dat=cr_pub0pubmed. 15767400 10.1128/JVI.79.7.3979-3986.2005 PMC1061563

[43] R. Tandon , V. Cattori , B. Willi , M. L. Meli , M. A. Gomes‐Keller , H. Lutz , et al., “Copy Number Polymorphism of Endogenous Feline Leukemia Virus‐Like Sequences.” Molecular and Cellular Probes 2007;21(4):257–266, 10.1016/j.mcp.2007.01.003. 17329079

[44] W. P. Maddison and L. L. Knowles , “Inferring Phylogeny Despite Incomplete Lineage Sorting.” Systematic Biology 2006;55(1):21–30, 10.1080/10635150500354928. 16507521

[45] B. McClintock , “The Significance of Responses of the Genome to Challenge.” Science 1984;226(4676):792–801, 10.1126/science.15739260. 15739260

[46] O. Jarrett , “Feline Leukaemia Virus.” In Practice 1979;1(2):15–23, 10.1136/inpract.1.2.15. 2993169

[47] P. Aiewsakun , L. Richard , A. Gessain , A. Mouinga‐Ondémé , P. Vicente Afonso , and A. Katzourakis , “Modular Nature of Simian Foamy Virus Genomes and Their Evolutionary History.” Virus Evolution 2019;5(2), vez032, 10.1093/ve/vez032. 31636999 PMC6795992

[48] J. Ottenburghs , “Ghost Introgression: Spooky Gene Flow in the Distant Past.” BioEssays 2020, 42(6): e2000012, 10.1002/bies.202000012. 32227363

