# Supplementary material for: Evolutionary Dynamics of Endogenous Feline Leukemia Virus in the Felis Genus Through the Lens of Genomics
Source: Ann N Y Acad Sci. 2026 Apr 21;1558:e70278. doi: 10.1111/nyas.70278 (PMC13099115; doi:10.1111/nyas.70278)
Supplement: Supplementary file 3 — Supplementary Material: nyas70278‐sup‐0001‐SuppMat‐ST1.pdf [file NYAS-1558-0-s001.pdf]

Table S1: Identification of reference genomes used throughout the study

Taxon	Submitted GenBank assembly	Genome Assembly	Identifier for this study	Submission Date	Genome Size (Gb for animal, Kb for virus)	Scaffold N50 (Mb)	Scaffold L50	Assembly level	Sequencing Technology
Felis catus	GCA_018350175.1	F.catus_Fca126_mat1.0	Fca_126	May 13, 2021	2.4	148.5	7	Chromosome	PacBio Sequel 2
Felis catus	GCA_000003115.1	catChrV17e	Fca_V17	Mar 16, 2009	3.2	16.3	47	Chromosome	ABI
Felis catus	GCA_016509815.2	Fcat_Pben_1.1_maternal_alt	Fca_Pb1.1	Jan 6, 2021	2.4	147.6	7	Chromosome	PacBio
Felis catus	GCA_000181335.6	felCat9.2_X	Fca_9.2	Oct 1, 2021	2.5	88.9	11	Chromosome	RSII; Sequel
Felis nigripes	GCA_032458615.1	SNNU_BFC_1	Fni_SNNU	Oct 11, 2023	2.4	146.9	7	Chromosome	PacBio Sequel II
Felis nigripes	GCA_028533295.1	Felis_nigripes_HiC	Fni_HiC	Feb 9, 2023	2.5	139.7	7	Chromosome	Illumina NovaSeq 6000
Felis chaus	GCA_019924945.1	FelChav1.0	Fch_1.0	Sep 14, 2021	2.4	148.6	7	Chromosome	PacBio Sequel
Prionailurus bengalensis	GCA_016509475.2	Fcat_Pben_1.1_paternal_pri	Pbe_1.1	Jan 6, 2021	2.4	148.6	7	Chromosome	PacBio
Otocolobus manul	GCA_028564725.2	OtoMan_p1.0	Oma_1.0	Nov 27, 2023	2.5	151.9	7	Chromosome	Oxford Nanopore
Puma concolor	GCA_003327715.1	PumCon1.0	Pco_1.0	Jul 19, 2018	2.4	100.5	8	Scaffold	Illumina; Oxford Nanopore
Acinonyx jubatus	GCA_027475565.2	VMU_Ajub_asm_v1.0	Aju_1.0	Dec 28, 2022	2.4	144.4	7	Chromosome	PacBio
Herpailurus yagouaroundi	GCA_014898765.1	PumYag	Hya_pya	Oct 20, 2020	2.5	49.3	15	Scaffold	Illumina NovaSeq
Lynx canadensis	GCA_007474595.2	mLynCan4.pri.v2	Lca_pri	Jul 14, 2020	2.4	147.3	7	Chromosome	PacBio Sequel I; Bionano Genomics; Arima Genomics Hi-C
Leopardus geoffroyi	GCA_018350155.1	O.geoffroyi_Oge1_pat1.0	Lge_1.0	May 13, 2021	2.4	152.6	7	Chromosome	PacBio Sequel 2
Caracal caracal	GCA_016801355.1	CarCar1.0	Cca_1.0	Feb 2, 2021	2.4	2.1	333	Scaffold	Illumina HiSeq
Panthera tigris	GCA_018350195.2	P.tigris_Pti1_mat1.1	Pti_1.1	May 13, 2021	2.4	146.9	7	Chromosome	PacBio Sequel 2
Neofelis nebulosa	GCA_028018385.1	mNeoNeb1.pri	Nne_pri	Jan 31, 2023	2.5	150.2	7	Chromosome	PacBio Sequel II HiFi; Bionano Genomics DLS; Arima Hi-C v2
Walleye dermal sarcoma virus	*NC_001867.1*	Walleye dermal sarcoma virus, complete genome	NC_001867. 1_|Walleye_dermal_sarcoma_virus	Aug 13, 2018	12.7	NA	NA	NA	NA
Feline leukemia virus	*NC_001940.1*	Feline leukemia virus, complete genome	NC_001940.1_|Feline_leukemia_virus	Aug 13, 2018	8.4	NA	NA	NA	NA
Feline leukemia virus	*OR682571.1*	Feline leukemia virus isolate FeLV/Lynx/DE/2020, complete genome	OR682571. 1_|Feline_leukemia_virus_Lynx2020	Nov 12, 2023	8.4	NA	NA	NA	NA
Feline leukemia virus	*LC765227.1*	Feline leukemia virus FeLV-A_ON-C_30 proviral DNA, complete genome	LC765227.1_|Feline_leukemia_virus	May 17, 2023	8.0	NA	NA	NA	NA
Feline leukemia virus	*MH116005.1*	Feline leukemia virus isolate FeLV_US_x2655_Fca2018, complete genome	MH116005. 1_|Feline_leukemia_virus_Fca2018	Dec 31, 2018	8.4	NA	NA	NA	NA
Feline leukemia virus	*MF681664.1*	Feline leukemia virus isolate FeLV_US_x1613_Fca2011, complete genome	MF681664. 1_|Feline_leukemia_virus_Fca2011	Dec 20, 2018	8.4	NA	NA	NA	NA
Feline leukemia virus	*MF681665.1*	Feline leukemia virus isolate FeLV_US_x2004_Pco2010, complete genome	MF681665. 1_|Feline_leukemia_virus_Pco2010	Dec 20, 2018	8.4	NA	NA	NA	NA
Feline leukemia virus	*MF681666.1*	Feline leukemia virus isolate FeLV_US_x2004R1_Pco2012, complete genome	MF681666. 1_|Feline_leukemia_virus_Pco2012	Dec 20, 2018	8.4	NA	NA	NA	NA
Friend murine leukemia virus	*NC_001362.1*	Friend murine leukemia virus FB29 complete genome	NC_001362. 1_|Friend_murine_leukemia_virus	Aug 13, 2018	8.3	NA	NA	NA	NA
Gibbon ape leukemia virus	*NC_001885.3*	Gibbon ape leukemia virus gag, pol, and env genes, complete cds	NC_001885.3_|Gibbon_ape_leukemia_virus	Aug 13, 2018	8.1	NA	NA	NA	NA
Koala retrovirus	*NC_039228.1*	Koala retrovirus gag protein (gag), pol protein (pol), and envelope protein (env) genes, complete cds	NC_039228.1_|Koala_retrovirus	Aug 24, 2018	8.4	NA	NA	NA	NA
Moloney murine leukemia virus	*NC_001501.1*	Moloney murine leukemia virus, complete genome	NC_001501. 1_|Moloney_murine_leukemia_virus	Aug 13, 2018	8.3	NA	NA	NA	NA
Moloney murine sarcoma virus	*NC_001502.1*	Moloney murine sarcoma virus, complete genome	NC_001502. 1_|Moloney_murine_sarcoma_virus	Aug 13, 2018	5.8	NA	NA	NA	NA
RD114 retrovirus	*NC_009889.1*	RD114 retrovirus, complete genome	NC_009889.1_|RD114_retrovirus	Aug 13, 2018	8.4	NA	NA	NA	NA
Jaagsiekte sheep retrovirus	*NC_001494.1*	Jaagsiekte sheep retrovirus, complete genome	NC_001494. 1_|Jaagsiekte_sheep_retrovirus_Betaretrovir us	Aug 13, 2018	7.5	NA	NA	NA	NA
Human T-lymphotropic virus 4	*NC_011800.1*	Human T-lymphotropic virus 4, complete genome	NC_011800.1_|Human_T-lymphotropic_virus_4_Deltaretrovirus	Aug 13, 2018	8.8	NA	NA	NA	NA
Feline foamy virus	*NC_039242.1*	Feline foamy virus DNA, complete genome	NC_039242. 1_|Feline_foamy_virus_Spumaretrovirinae	Aug 24, 2018	11.7	NA	NA	NA	NA
Avian leukemia virus	*NC_015116.1*	Avian leukemia virus isolate SCDY1, complete genome	NC_015116. 1_|Avian_leukemia_virus_alpharetrovirus	Aug 13, 2018	7.5	NA	NA	NA	NA
Feline immunodeficiency virus	*NC_001482.1*	Feline immunodeficiency virus complete genome	NC_001482. 1_|Feline_immunodeficiency_virus_Lentivir us	Aug 13, 2018	9.5	NA	NA	NA	NA
