# Supplementary material for: Evolutionary Dynamics of Endogenous Feline Leukemia Virus in the Felis Genus Through the Lens of Genomics
Source: Ann N Y Acad Sci. 2026 Apr 21;1558:e70278. doi: 10.1111/nyas.70278 (PMC13099115; doi:10.1111/nyas.70278)
Supplement: Supplementary file 4 — Supplementary Material: nyas70278‐sup‐0001‐SuppMat‐ST2.pdf [file NYAS-1558-0-s004.pdf]

Table S2: Content summary of enFeLV copies

Genomic Signature	Taxon	Genome Assembly	Chromosome	Length (nt)	Dating (years)	Gag gene			Pol gene			Env gene			Recombination Evidence
						Presence	Stop Codons (frame)	600 nt ORF	Presence	Stop Codons (frame)	600 nt ORF	Presence	Stop Codons (frame)	600 nt ORF	
AAAC	*Felis catus*	Fcat_Pben_1.1_maternal_alt	D1	4221	NA	T	7 (1/3)	2	F	NA	0	T	13 (1)	1	F
ACAG	*Felis catus*	F.catus_Fca126_mat1.0	B2	8673	NA	T	0 (2)	1	T	0 (2)	1	T	17 (1)	2	F
AGCC	*Felis catus*	Fcat_Pben_1.1_maternal_alt	C1	4221	NA	T	4 (2)	2	F	NA	0	T	18 (2)	1	F
ATAC	*Felis catus*	felCat9.2_X	D4	8749	6,880,936	T	5 (1)	1	T	34 (2)	1	T	20 (3)	0	T
ATAC	*Felis catus*	Fcat_Pben_1.1_maternal_alt	D4	8669	NA	T	0 (3)	1	T	0 (1)	1	T	0 (3)	1	F
ATAG	*Felis catus*	Fcat_Pben_1.1_maternal_alt	D3	4222	348,385	T	4 (2)	2	F	NA	0	T	13 (2)	1	F
ATGG	*Felis catus*	Fcat_Pben_1.1_maternal_alt	B2	8604	348,385	T	4 (3)	2	T	0 (2)	1	T	0 (1)	1	F
ATGG	*Felis catus*	felCat9.2_X	B2	8653	2,828,187	T	0 (1)	1	T	38 (1/2)	2	T	17 (1)	2	F
ATGG	*Felis catus*	F.catus_Fca126_mat1.0	B2	8604	NA	T	0 (1)	1	T	0 (3)	1	T	0 (2)	1	F
CTAA	*Felis catus*	felCat9.2_X	B1	4181	4,278,458	T	2 (2)	0	F	NA	0	T	20 (2)	1	T
CTAG	*Felis catus*	Fcat_Pben_1.1_maternal_alt	A1	4714	NA	T	3 (3)	1	F	0 (2)	1	T	16 (2)	0	T
CTCT	*Felis catus*	Fcat_Pben_1.1_maternal_alt	B4	8681	697,610	T	6 (3)	1	T	1 (3)	2	T	18 (2)	2	F
CTGT	*Felis catus*	F.catus_Fca126_mat1.0	B4	8670	348,385	T	0 (2)	1	T	0 (3)	1	T	0 (2)	1	F
CTGT	*Felis catus*	felCat9.2_X	B4	8679	NA	T	0 (3)	1	T	22 (3)	2	T	0 (2)	1	T
CTTG	*Felis catus*	F.catus_Fca126_mat1.0	D4	8704	NA	T	5 (3)	2	T	1 (2)	1	T	1 (2)	2	T
GGAG	*Felis catus*	Fcat_Pben_1.1_maternal_alt	B4	8702	NA	T	0 (2)	1	T	1 (2)	1	T	0 (1)	1	F
GGAG	*Felis catus*	F.catus_Fca126_mat1.0	B4	8702	NA	T	0 (2)	1	T	1 (2)	1	T	0 (1)	1	F
GTTC	*Felis catus*	Fcat_Pben_1.1_maternal_alt	B1	4509	697,610	T	3 (1)	2	F	0 (1)	1	T	0 (3)	1	F
GTTC	*Felis catus*	F.catus_Fca126_mat1.0	B1	4512	1,049,378	T	3 (1)	2	F	0 (1)	1	T	0 (3)	1	F
GTTC	*Felis catus*	felCat9.2_X	B1	4520	3,550,459	T	20 (3)	0	F	5 (2)	0	T	7 (2)	0	F
GTTT	*Felis catus*	Fcat_Pben_1.1_maternal_alt	B3	8672	NA	T	0 (2)	1	T	0 (1)	1	T	0 (1)	1	F
GTTT	*Felis catus*	F.catus_Fca126_mat1.0	B1	4196	NA	T	5 (2)	0	F	NA	0	T	18 (1)	0	F
TGAC	*Felis catus*	Fcat_Pben_1.1_maternal_alt	B4	4523	1,048,526	T	0 (2)	2	F	0 (2)	1	T	0 (1)	1	F
TGAC	*Felis catus*	catChrV17e	B1	4517	1,401,435	T	0 (2)	1	F	1 (1)	1	T	0 (3)	1	F
TGTT	*Felis catus*	felCat9.2_X	F1	5477	9,592,511	T	17 (2)	0	F	4 (3)	0	T	20 (2)	0	T
TGTT	*Felis catus*	Fcat_Pben_1.1_maternal_alt	F1	5562	NA	T	6 (1)	2	F	0 (1)	1	T	17 (1)	0	T
TTAT	*Felis catus*	felCat9.2_X	X	8890	9,970,284	T	15 (3)	1	T	41 (2)	1	T	13 (2)	1	T
AAAG	*Felis chaus*	FelChav1.0	C1	7058	NA	T	0 (1)	1	T	5 (2)	1	T	0 (1)	1	F
AAAG	*Felis chaus*	FelChav1.0	D2	8397	NA	T	0 (2)	1	T	0 (2)	1	T	13 (1)	2	F
ACCT	*Felis chaus*	FelChav1.0	D2	8693	NA	T	0 (3)	1	T	0 (3)	1	T	0 (1)	1	F
ACGT	*Felis chaus*	FelChav1.0	D4	8547	NA	T	0 (1)	1	T	0 (1)	1	T	0 (3)	1	F
ACTT	*Felis chaus*	FelChav1.0	A1	4839	NA	T	0 (1)	1	T	NA	0	T	0 (1)	1	F
AGAT	*Felis chaus*	FelChav1.0	A2	8688	NA	T	0 (2)	1	T	0 (2)	1	T	0 (1)	1	F
ATAC	*Felis chaus*	FelChav1.0	C1	4750	NA	T	0 (1)	1	T	NA	0	T	0 (1)	1	F
ATAT	*Felis chaus*	FelChav1.0	B3	4717	NA	T	8 (2)	2	T	NA	0	T	0 (3)	1	F
ATCA	*Felis chaus*	FelChav1.0	D1	6931	NA	T	0 (2)	1	T	0 (1)	1	T	0 (3)	1	F
ATCT	*Felis chaus*	FelChav1.0	F1	7065	NA	T	0 (1)	1	T	5 (2)	1	T	0 (1)	1	F
ATTC	*Felis chaus*	FelChav1.0	D4	7047	NA	T	0 (3)	1	T	0 (1)	1	T	1 (3)	1	F
CAGG	*Felis chaus*	FelChav1.0	B3	8690	348,385	T	0 (1)	1	T	0 (1)	1	T	0 (3)	1	F
CCAG	*Felis chaus*	FelChav1.0	B2	4850	NA	T	0 (1)	1	T	NA	0	T	0 (1)	1	F
CCAG	*Felis chaus*	FelChav1.0	B2	8672	NA	T	0 (2)	1	T	0 (2)	1	T	0 (1)	1	F
CCAT	*Felis chaus*	FelChav1.0	C2	7073	NA	T	0 (3)	1	T	0 (2)	1	T	8 (2)	1	F
CTCT	*Felis chaus*	FelChav1.0	F1	7058	NA	T	6 (2)	2	T	0 (1)	1	T	0 (3)	1	F
CTGG	*Felis chaus*	FelChav1.0	D1	7040	NA	T	0 (2)	1	T	0 (1)	1	T	10 (2)	2	F
CTGT	*Felis chaus*	FelChav1.0	B1	6754	348,106	T	0 (3)	1	T	0 (1)	1	T	7 (3)	2	F
CTGT	*Felis chaus*	FelChav1.0	C1	8659	NA	T	0 (1)	1	T	0 (1)	1	T	12 (2)	2	F
GAAT	*Felis chaus*	FelChav1.0	B3	6768	348,385	T	0 (3)	1	T	0 (1)	1	T	13 (3)	2	F
GCAT	*Felis chaus*	FelChav1.0	E2	4054	NA	F	0 (2)	1	F	0 (2)	1	T	0 (1)	1	F
GCGA	*Felis chaus*	FelChav1.0	B2	4819	NA	T	0 (3)	1	T	NA	0	T	0 (3)	1	F
GGTG	*Felis chaus*	FelChav1.0	C1	7052	NA	T	0 (1)	1	T	0 (3)	1	T	0 (2)	1	F
GTTT	*Felis chaus*	FelChav1.0	A1	4838	NA	T	0 (2)	1	T	NA	0	T	1 (2)	1	F
TCCT	*Felis chaus*	FelChav1.0	Y	8683	NA	T	0 (1)	1	T	0 (1)	1	T	0 (3)	1	F
TTGG	*Felis chaus*	FelChav1.0	B2	4836	NA	T	0 (3)	1	T	NA	0	T	13 (2)	2	F
TTTT	*Felis chaus*	FelChav1.0	C1	4738	NA	T	0 (2)	1	T	NA	0	T	0 (2)	1	F
AGGT	*Felis nigripes*	SNNU_BFC_1	B4	5812	348,385	T	7 (2)	1	F	12 (1)	1	T	14 (1)	1	F
ATAA	*Felis nigripes*	SNNU_BFC_1	A2	8609	348,385	T	0 (2)	1	T	0 (1)	1	T	17 (3)	2	F
CACT	*Felis nigripes*	SNNU_BFC_1	C1	6386	NA	T	6 (2)	2	F	10 (1)	1	T	7 (2)	1	F
CAGG	*Felis nigripes*	SNNU_BFC_1	A1	8681	NA	T	1 (2)	1	T	34 (1)	3	T	0 (2)	1	F
CCAG	*Felis nigripes*	SNNU_BFC_1	A1	6370	NA	T	8 (3)	2	F	10 (2)	1	T	2 (1)	1	F
CTAC	*Felis nigripes*	SNNU_BFC_1	C1	6139	348,106	T	6 (2)	2	F	10 (2)	1	T	18 (2)	1	F
CTCT	*Felis nigripes*	SNNU_BFC_1	E1	8701	348,385	T	10 (3)	2	T	13 (2)	2	T	0 (1)	1	F
CTGT	*Felis nigripes*	SNNU_BFC_1	F2	8694	NA	T	4 (1)	2	T	0 (2)	1	T	1 (1)	2	F
GCTC	*Felis nigripes*	SNNU_BFC_1	B2	6388	699,017	T	2 (3)		F	10 (2)	1	T	18 (1)	1	F
GGTG	*Felis nigripes*	SNNU_BFC_1	E2	6362	697,610	T	2 (1)	1	F	10 (3)	1	T	19 (3)	1	F
TTTG	*Felis nigripes*	SNNU_BFC_1	D4	2959	1,049,378	F	NA	1	F	NA	0	T	0 (2)	1	F
