# Supplementary material for: Evolutionary Dynamics of Endogenous Feline Leukemia Virus in the Felis Genus Through the Lens of Genomics
Source: Ann N Y Acad Sci. 2026 Apr 21;1558:e70278. doi: 10.1111/nyas.70278 (PMC13099115; doi:10.1111/nyas.70278)
Supplement: Supplementary file 1 — Supplementary Material: nyas70278‐sup‐0001‐SuppMat‐SF1.pdf [file NYAS-1558-0-s002.pdf]

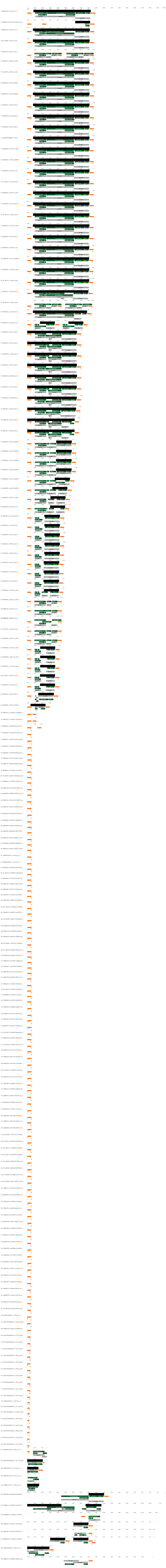

**Figure S1:** Annotation of genes and peptides of each enFeLV copy. Annotations were based on global similarity higher than 25% and local similarity according to manual trimming of pairwise alignments. Types of annotations are separated in a color scheme: in orange, LTR regions according to the reference enFeLV sequence AY364319; in grey, retroviral genes according to the reference enFeLV sequence AY364319; in green, retroviral peptides according to the reference exogenous FeLV sequence NC_001940.1. All analyses were performed using Geneious software v.2022.2.2.
