# Supplementary material for: Evolutionary Dynamics of Endogenous Feline Leukemia Virus in the Felis Genus Through the Lens of Genomics
Source: Ann N Y Acad Sci. 2026 Apr 21;1558:e70278. doi: 10.1111/nyas.70278 (PMC13099115; doi:10.1111/nyas.70278)
Supplement: Supplementary file 2 — Supplementary Material: nyas70278‐sup‐0001‐SuppMat‐SF2.pdf [file NYAS-1558-0-s003.pdf]

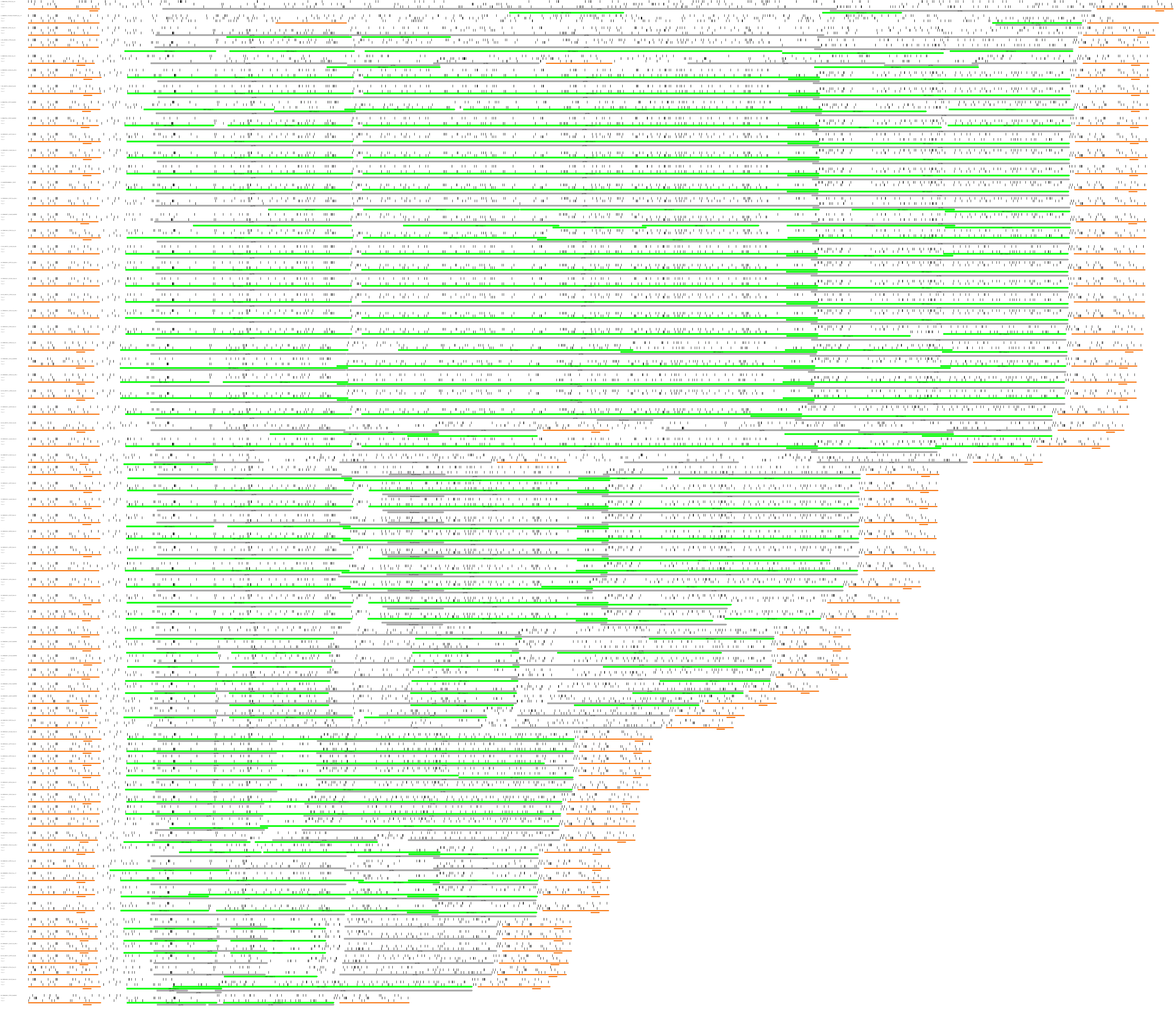

Figure S2: Visualization of stop codons and open reading frames (ORFs) of each enFeLV copy. The annotation of genes (grey) and LTR regions (orange) were included in order to contextualize the sequences; they were determined according to a 25% or higher global similarity to the reference enFeLV sequence AY364319 and local similarity according to manual trimming of pairwise alignments. ORFs longer than 600 nucleotides are represented in green and stop codons are represented as black stripes vertically separated by reading frames. All analyses were performed using Geneious software v.2022.2.2
